# Supplementary figures and images for: Prediction and expression analysis of deleterious nonsynonymous SNPs of Arabidopsis ACD11 gene by combining computational algorithms and molecular docking approach
Source: PLoS Comput Biol. 2022 Jun 16;18(6):e1009539. doi: 10.1371/journal.pcbi.1009539 (PMC9242461; doi:10.1371/journal.pcbi.1009539)

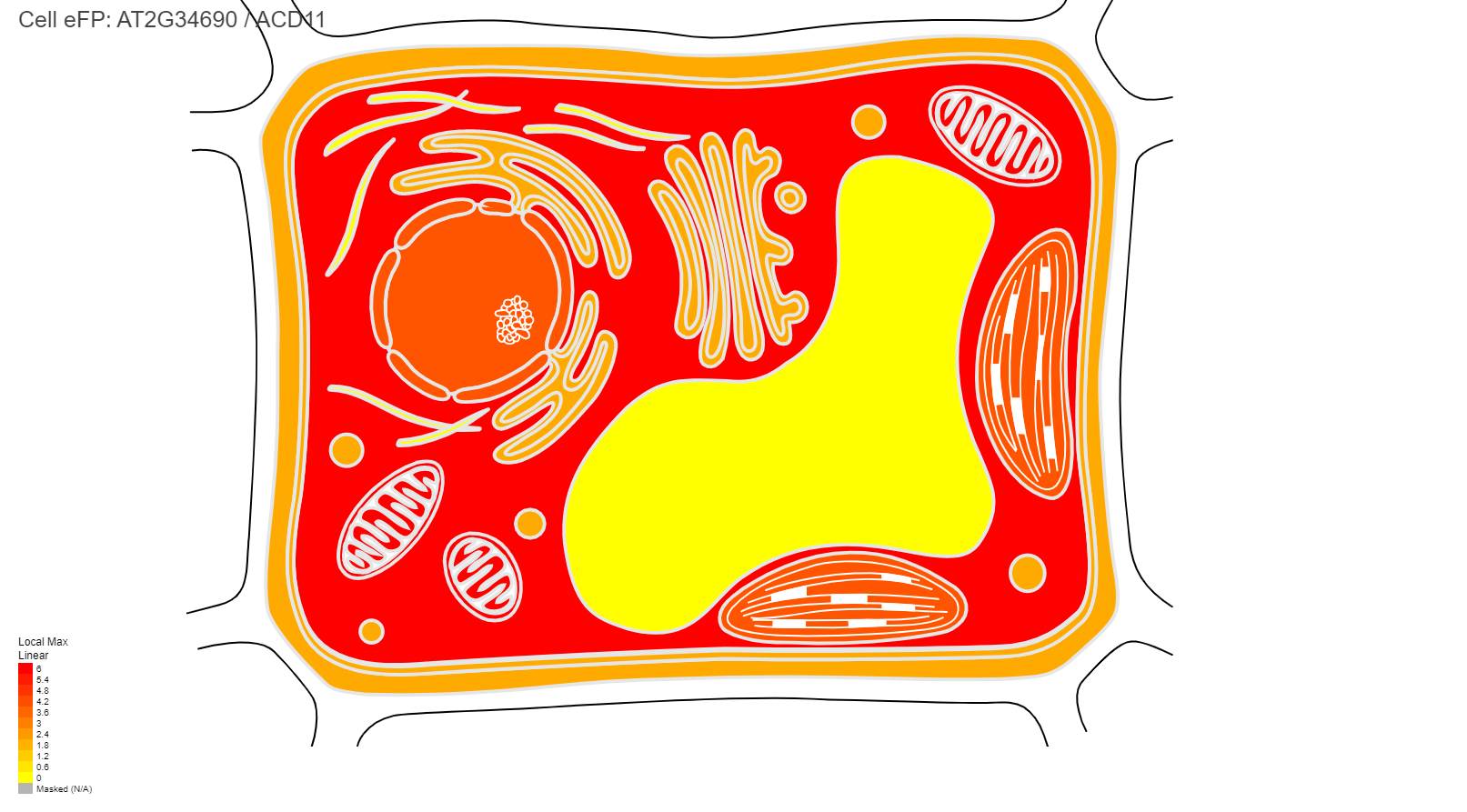

Supplement: S1 Fig — (PNG) [file pcbi.1009539.s001.png]

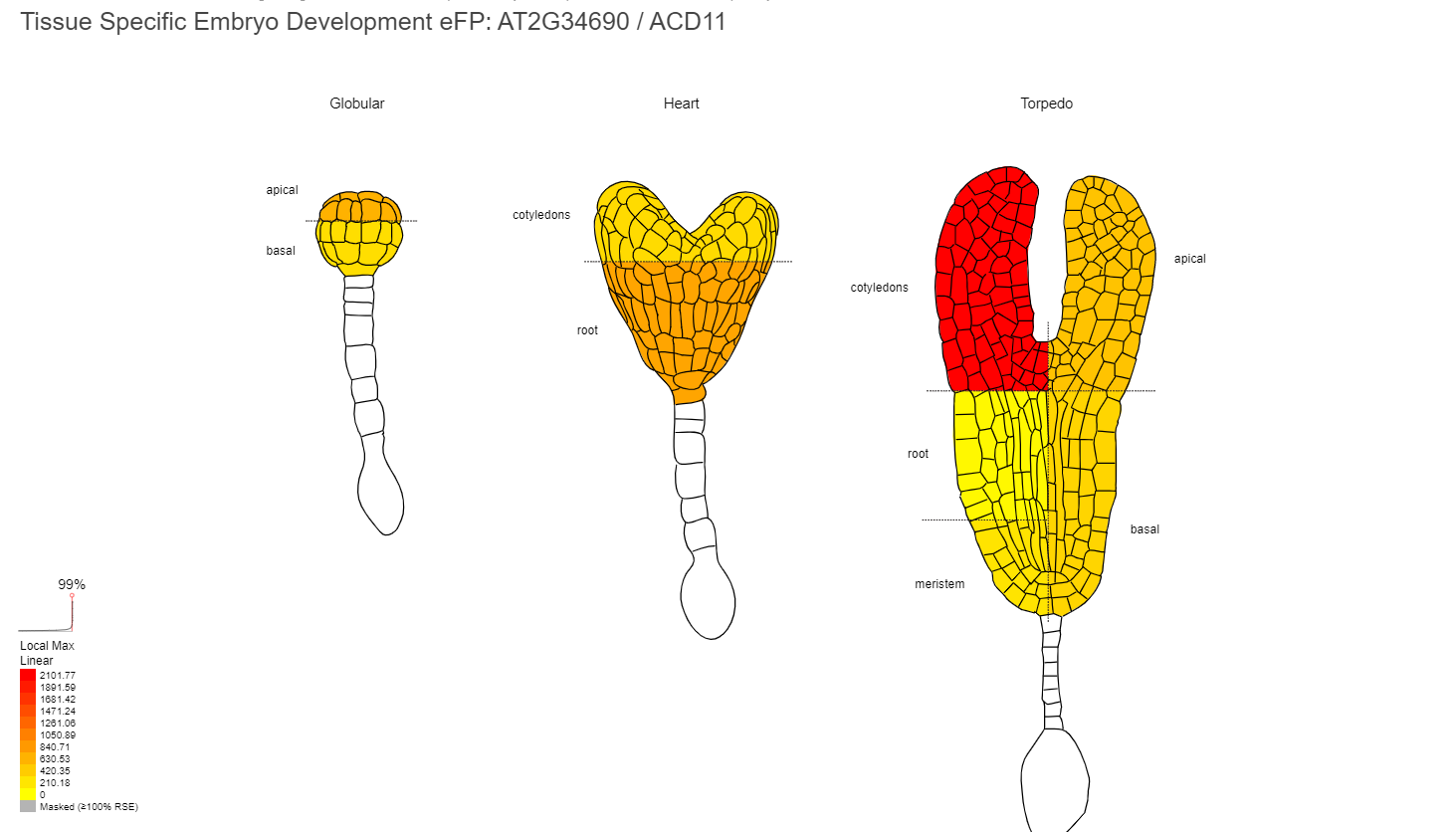

Supplement: S2 Fig — (PNG) [file pcbi.1009539.s002.png]

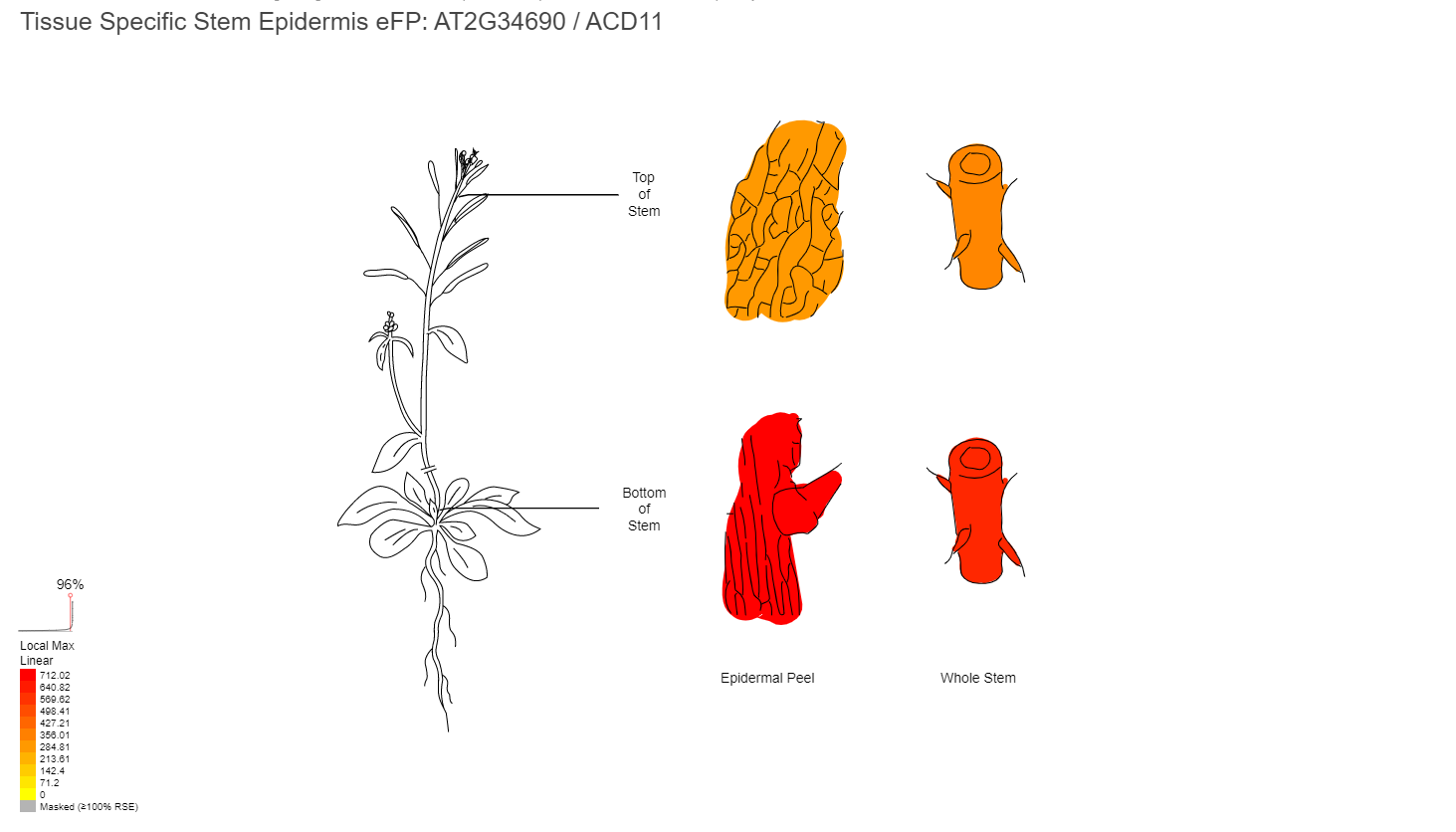

Supplement: S3 Fig — (PNG) [file pcbi.1009539.s003.png]

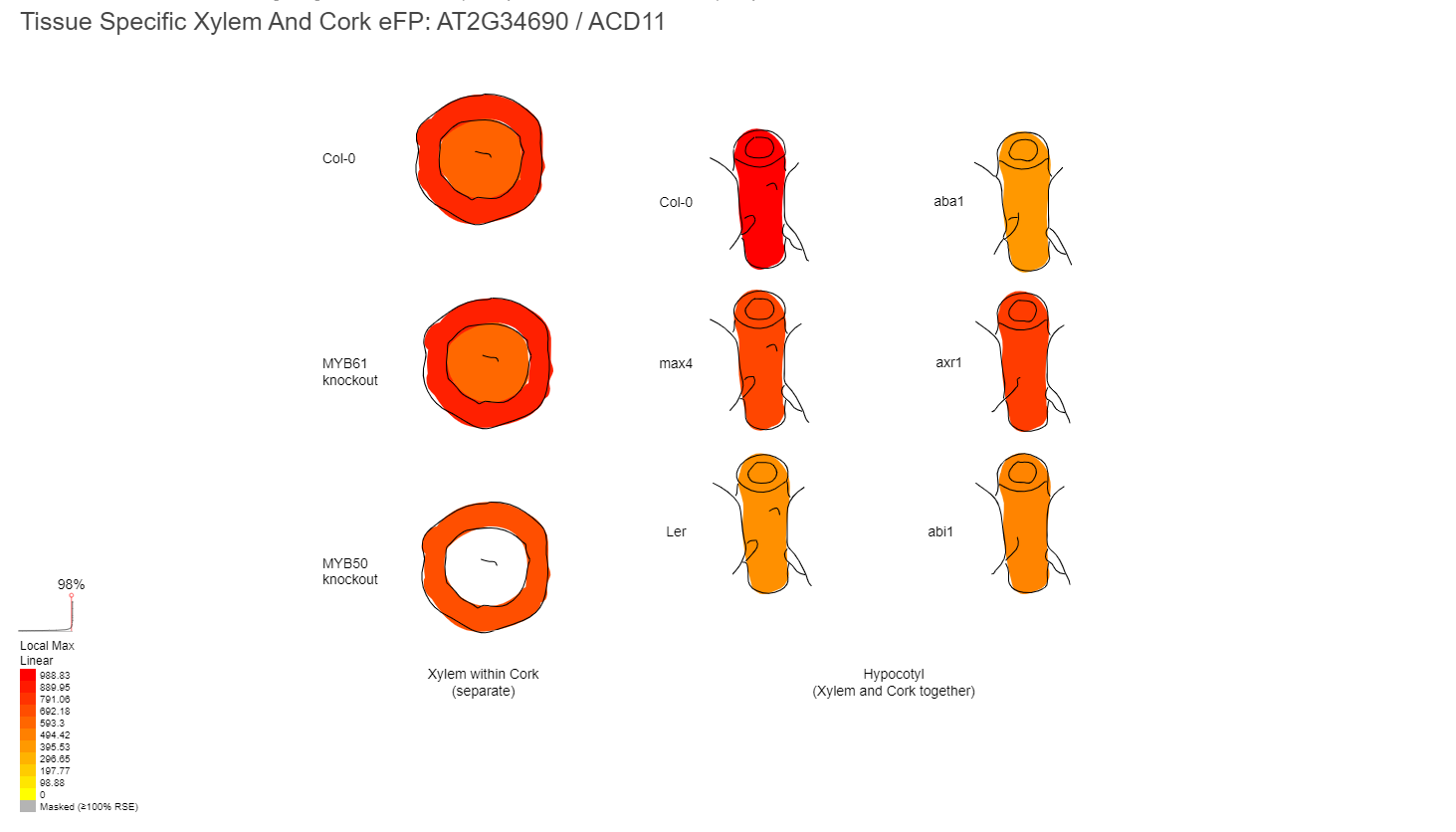

Supplement: S4 Fig — (PNG) [file pcbi.1009539.s004.png]

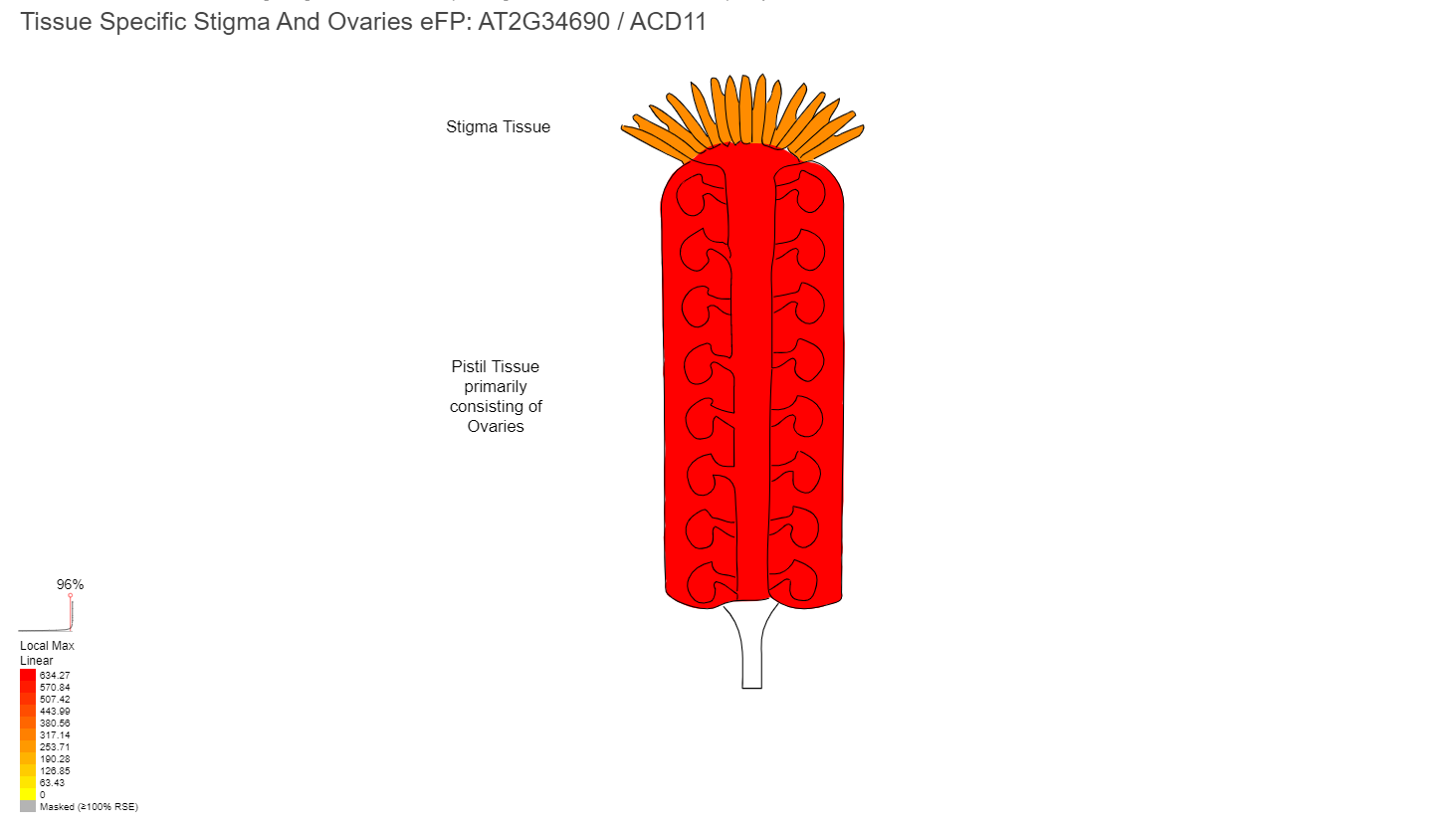

Supplement: S5 Fig — (PNG) [file pcbi.1009539.s005.png]

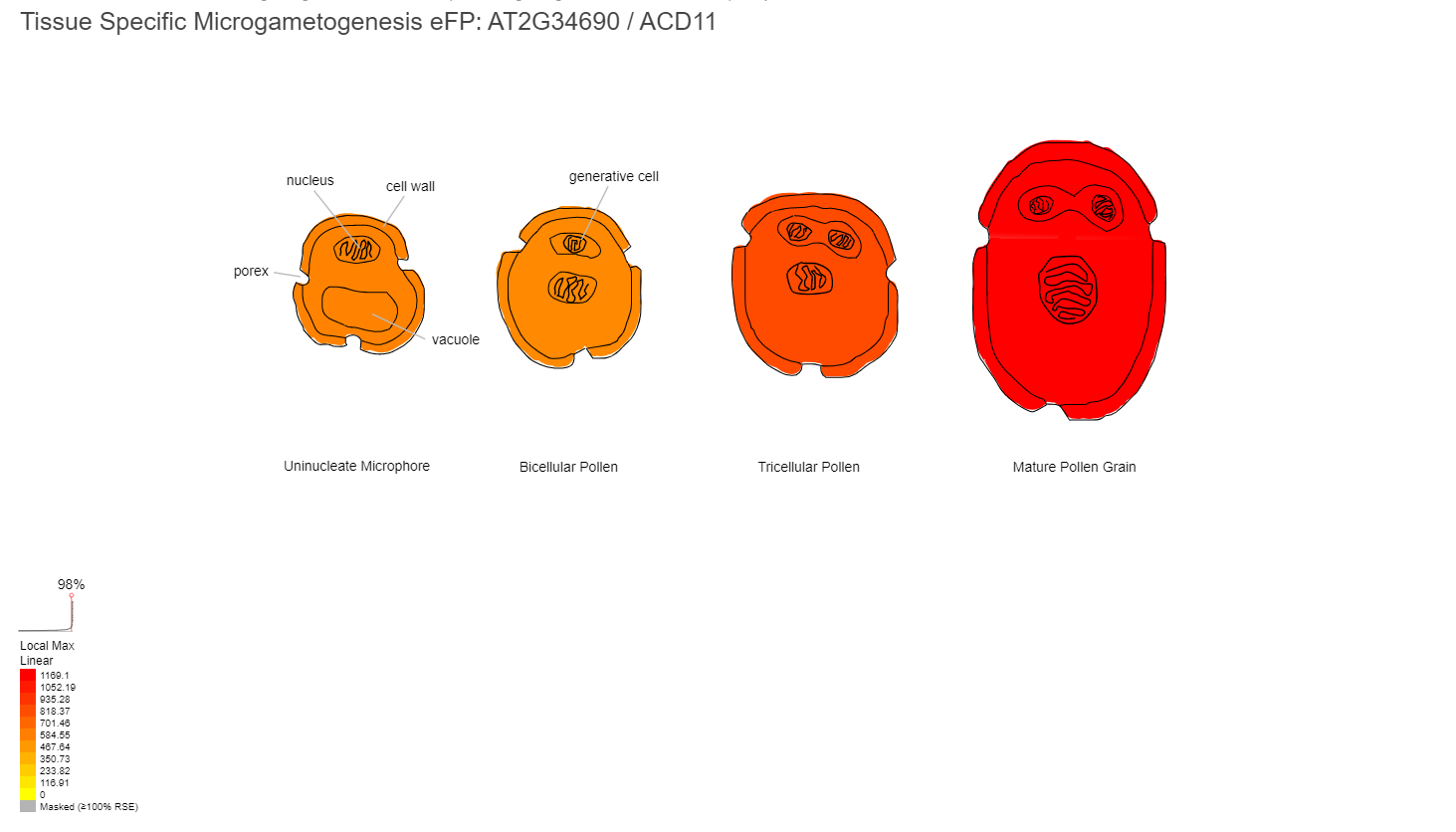

Supplement: S6 Fig — (PNG) [file pcbi.1009539.s006.png]

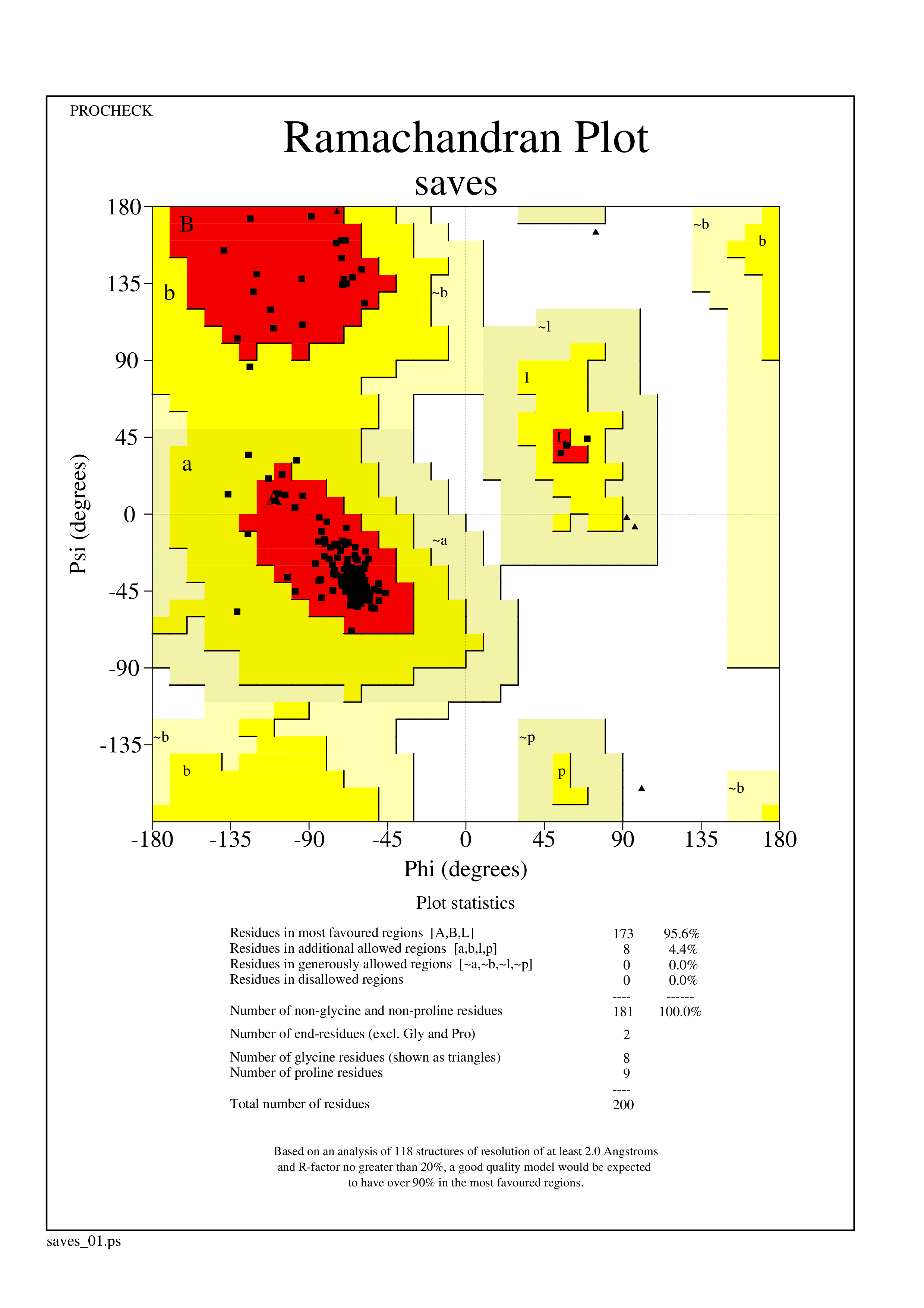

Supplement: S7 Fig — (PNG) [file pcbi.1009539.s007.png]

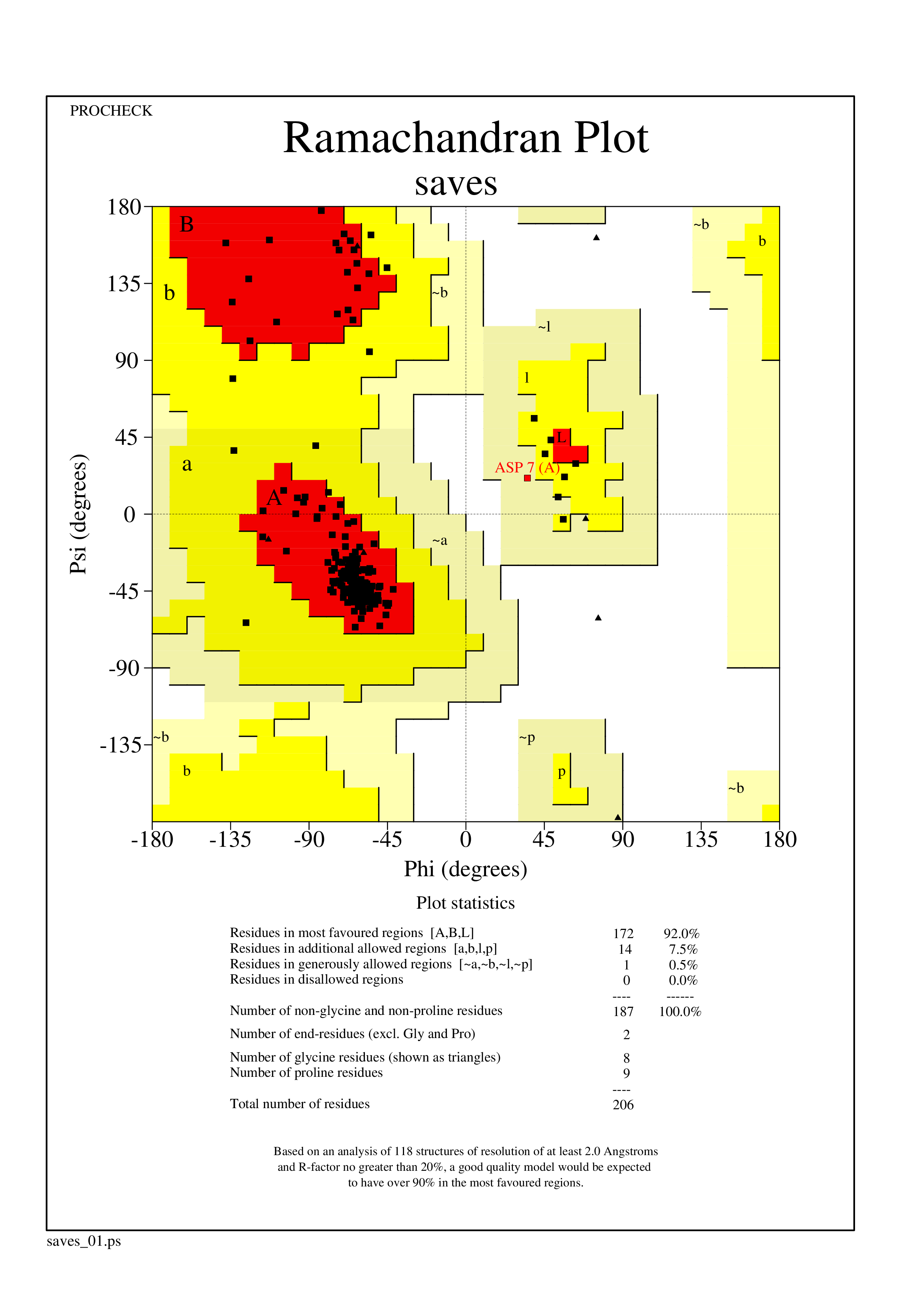

Supplement: S8 Fig — (PNG) [file pcbi.1009539.s008.png]

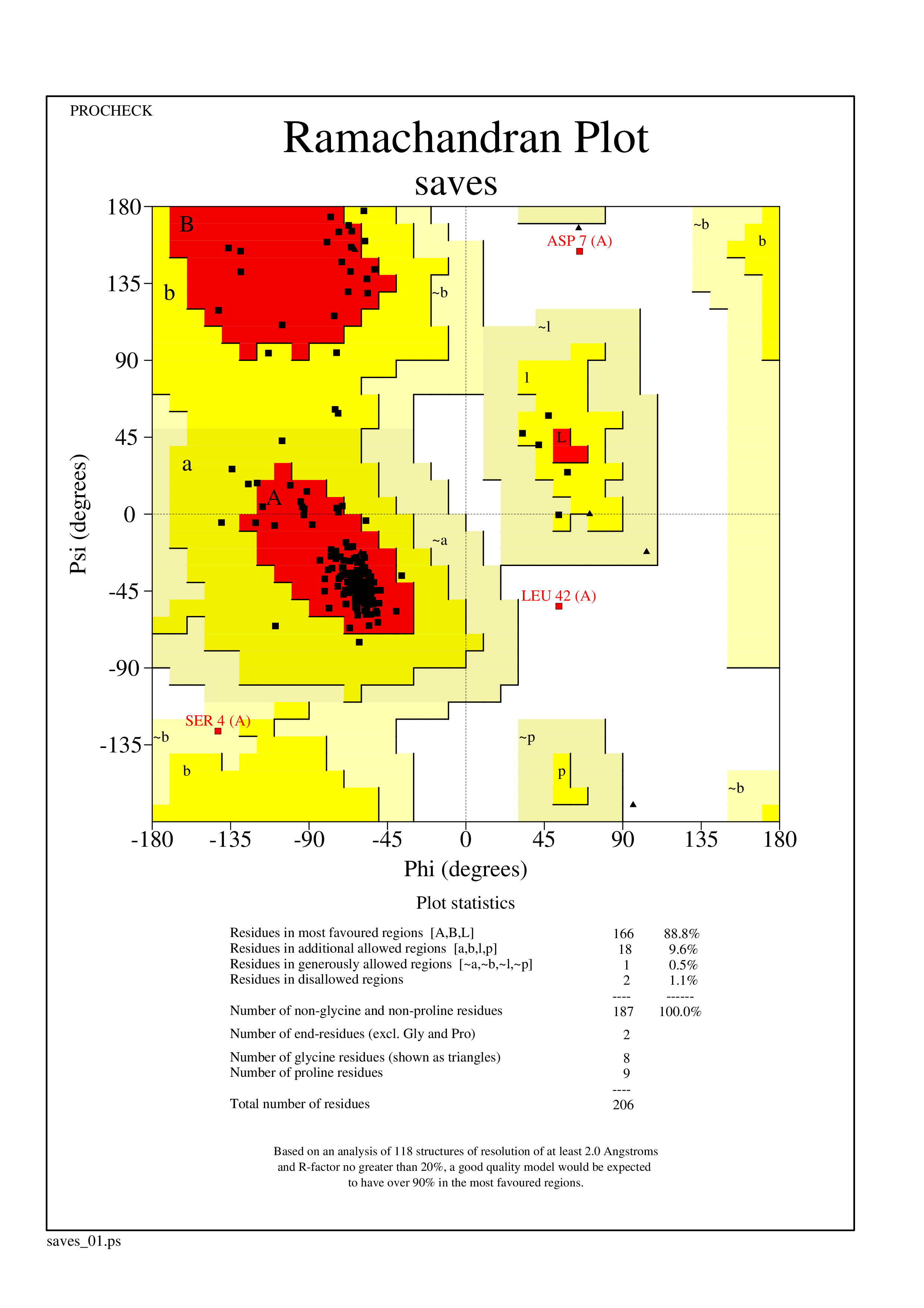

Supplement: S9 Fig — (PNG) [file pcbi.1009539.s009.png]
